# Clinically Significant Fatigue in Adult Leukemia Patients: Prevalence, Predictors, and Impact on Quality of Life

**DOI:** 10.7759/cureus.12245

**Published:** 2020-12-23

**Authors:** Isamme AlFayyad, Mohamad Al-Tannir, Muawiyah Yaqub, Humariyah Heena, Nawaf AlMukaibil, Mohammed Ghazwani, Amani Abu-Shaheen

**Affiliations:** 1 Epidemiology and Public Health, King Fahad Medical City, Riyadh, SAU; 2 Oncology, King Fahad Medical City, Riyadh, SAU; 3 Preventive Medicine, King Fahad Medical City, Riyadh, SAU; 4 Internal Medicine, Al-Imam Mohammad Ibn Saud Islamic University, Riyadh, SAU; 5 Internal Medicine, King Fahad Medical City, Riyadh, SAU

**Keywords:** quality of life (qol), leukemia, clinically significant fatigue, adults, cancer

## Abstract

Background

Cancer-related fatigue (CRF) is a common distressing symptom in leukemia patients. CRF becomes clinically significant fatigue (CSF) when adversely affects health-related quality of life (HRQoL) and warrants further workup, referrals, and treatment. Objective: To assess the prevalence and predictors of CSF and assesses its impact on HRQoL in adult leukemia patients.

Method

Analysis was performed on 168 leukemia patients. The primary study outcomes were CSF (score ≥4) as measured by the fatigue numerical rating scale and HRQoL using a validated Functional Assessment of Cancer Therapy-Leukemia (FACT-Leu) scale.

Result

The prevalence of CSF was 89 (53%), with a mean score of 6.66±2.02. About 106 (63.1%) of leukemia patients had poor Health-related quality of life (HRQoL) (102.61±23.50). Overall, FACT-Leu mean score indicated that study participants had poor HRQoL (114.70±29.67). There was a statistically significant difference in HRQoL between the patients with CSF 104.89±28.82 and Non-CSF 125.76±26.71, p<0.001. Poor appetite (odd ratio: 3.02 [95% CI: 1.33-6.85]) was statistically significant predictors (p<0.010) of CSF. Dependence on caregiver (odd ratio: 3.31 [95% CI: 0.41-0.75]) and having non-CSF (odd ratio: 5.22 [95% CI: 2.44-11.19]) were found statistically significant predictors of good HRQoL.

Conclusion

CSF is prevalent among leukemia patients, and adversely affects their HRQoL. Holistic assessment and supportive care are needed to reduce the burden of CSF and improve leukemia patients HRQoL.

## Introduction

Cancer-related fatigue (CRF) is the most prevalent, persistent, and distressing symptoms among cancer patients and survivors [1,2]. CRF is a debilitating symptom that interferes with physical and mental function and associated with reduced quality of life [3].

The prevalence of CRF is varied worldwide, reflecting the variety of the studied populations, the subjective nature of the cancer disease, and the treatment modalities, as well as the various used screening methods. CRF has been reported in patients with active chemotherapy between 59-90% and up to 100 in patients receiving radiotherapy and up to 25% among cancer survivors [4-7]. Among cancer diseases, CRF was experienced and frequently reported by leukemia patients than patients with solid tumors, with prevalence ranges between 33% and 69% [8,9]. Fatigue continues after the completion of the treatment course, roughly in a quarter of treated patients and increasing up to 35% of long-term cancer survivors [10]. The high prevalence of CRF indicates that symptom management is challenging and still not well managed in a remarkable proportion of cancer patients’ life [3].

The clinical practice guidelines for CRF issued by the National Comprehensive Cancer Network (NCCN) represent the best “standard of care information” for the screening, assessment, and management of CRF [11]. These guidelines classified CRF into four clinically relevant subgroups according to their severity ratings to ensure that CRF is identified promptly and treated effectively. Understanding the relationship between the 4-subgroups and its clinically meaningful outcomes is significant for clinicians to inform and direct treatment decisions, promote clinician-patient dialogue on Clinically Significant Fatigue (CSF) management, and development of clinical practice guidelines [10]. The prevalence of CSF depends on the threshold score of severity (usually defined if the CRF a score of ≥ 4) that is persistent, associated disability, warrants further workup, referrals, and treatment [2,11].

The existing literature reported that risk factors associated with CRF are multifactorial. Some evidence suggests that CRF is associated with anemia, cachexia, neurological changes, infection, metabolic and endocrine disorders, psychological distress, concomitant medications, anti-neoplastic side-effects, pain, and paraneoplastic neurological syndromes [12]. Life prolongation, alleviation of the distressing symptoms, and preserving the optimal quality of life of cancer patients are the ultimate goals of cancer care [13]. However, CSF is associated with reduced cancer patients’ health-related quality of life (HRQoL) at all stages of cancer and results in substantial adverse effects physical impairment, psychosocial distress, and economic burden for both patients and caregivers [14]. HRQoL data afford pertinent information for clinicians on the effectiveness of cancer treatment and are a crucial source of information for cancer patients to make informed decisions in their care plan [15]. 

As far as we know, there is a scarcity in the empirical evidence from developing countries on the prevalence of leukemia patients and its impact on HRQoL. Therefore, we aimed in this study to assess the prevalence and predictors of CSF and assess its impact of HRQoL in adult leukemia patients.

## Materials and methods

Study design and Setting

A cross-sectional study was conducted in the comprehensive cancer centre (CCC) at King Fahad Medical City (KFMC), the largest tertiary care medical health care institution at the Saudi Ministry of Health.

Study sample and sampling method

A convenient sample of leukemia patients attending the outpatients’ chemotherapy unit, hematology led clinic, or hospitalized as inpatient between May 2018 and January 2019 was approached by a trained study coordinator to participate in this study. We included patients aged 18 years and above, leukemia malignancy irrespective of their leukemia type, mentally competent, and free of preexisting psychiatric disorders. Patients with other hematological malignancies, unwilling to participate in the study were excluded.

Sample size

We used Cochran’s method for estimating sample size with parameters of 95% confidence interval, 50% assumed prevalence of CRF and population size of 300 leukemia patients (cases on active treatment and follow-up in the CCC at KFMC in 2017); it was calculated that a minimum of 168 participants is required for this study.

Survey tools

The CRF was assessed using a numeric rating scale (0-10 points). We assessed the CRF severity as defined by the NCCN practice guidelines for cancer-related fatigue (0; no fatigue, 1-3; mild fatigue, 4-6; moderate fatigue, 7-10 severe fatigue), and CSF when the patient has fatigue ≥4 [16]. HRQoL was evaluated using the Functional Assessment of Cancer Therapy-Leukemia (FACT-Leu), a 44-items measure of HRQoL and Leukemia specific symptoms utilizing essential customized questions of the Functional Assessment of Cancer Therapy-General (FACT-G), along with a cancer site-specific Leukemia subscale [17]. The FACT-G consists of 4 primary domains: Physical Well-being (PWB) (7-items; score range 0 to 28), Social/Family Well-being (SWB) (7-items; score range 0 to 28), Emotional Well-being (EWB) (6-items; score range 0 to 24), Functional Well-being (FWB) (7-items; score range 0 to 28). The additional Leukemia-Specific Subscale (LEUS) comprised of 17-items (score range o to 68) to evaluate specific concerns related to Leukemia [17].

We used the theoretical bio-psychosocial model adapted Hwang et al., to examine the effect of the socio-demographic characteristics variables, clinical relevant conditions variables, and biomedical parameters variables and their possible association as predictors for the study outcomes [18]. The examined socio-demographic characteristics included (age, gender, marital status, employment status, and need for caregiver), clinical relevant conditions (underlying chronic illness, time since diagnosis, relapsed disease, type of cancer treatment, sleeping hours, weight loss per kilogram within last 6-months, Eastern Co-operative Oncology Group Performance Status (ECOG-PS), dehydration, infection, and poor appetite), and biomedical parameters including Haemoglobin level (Hb), White Blood Cells (WBCs), sodium, Creatinine level, Calcemia, Potassium, Aspartate Transaminase (AST), total Bilirubin, Alanine Aminotransferase (ALT), Lactate Dehydrogenase (LDH). The cut-off values for the biomedical parameters (as approved by the laboratory lab at the study site) are described in table 1.

Data analysis

Statistical analyses were done with IBM SPSS (Statistical Product and Service Solutions) Statistics (Version 24.0, Chicago). Descriptive analyses were used to summarize patients’ characteristics (socio-demographic, clinical relevant conditions, biomedical parameters) and FACT-Leu Scale and subscale (means ants standard deviations). Univariate and multivariate regression models were developed to identify independent predictors of CSF and FACT-Lue. If the p-value was <0.05 in univariate models, the possible predictors were used in multiple logistic regression models. An independent sample t-test was used to compare the difference in the mean score of FACT-Leu scale and domains. Receiver Operating Characteristic (ROC) was run to determine the optimal cut-off score for good HRQoL.

Ethical approval

Approval for the study was granted from the institutional review board at KFMC. Written informed consent was obtained before patients participated in the study.

## Results

One hundred and sixty-eight participants had completed the study survey. The study participants had an average of 43.99±19.20 years of age. Most of the participants were males 103 (61.3%), married 98 (58.7%), and dependent on caregiver 110 (65.5%). The mean time after diagnosis averaged 28.37±29.17 months, and the mean for weight loss (kg) averaged -1.14±6.92. The majority of the participants had chronic illnesses 107 (63.7%) and on active treatment 127 (75.6%). Poor appetite and sleeping < 7 hours was indicated by 101 (60.1%) and 71 (42.3%), respectively. The mean haemoglobin level and WBCs were 11.08±2.22 and 5.79±4.20 (Table 1).

**Table 1:** Bio-demographics of the study participants (n=168). ECOG-PS- Eastern Co-operative Oncology Group performance status; AST- aspartate transaminase, ALT- alanine aminotransferase, LDH- lactate dehydrogenase

Socio-demographic characteristics	
Age (mean ±SD)	43.99±19.20
Gender	
Male	103 (61.3%)
Female	65 (38.7%)
Marital Status	
Single	49 (29.3%)
Married	98 (58.7%)
Divorced	8 (4.8%)
Widowed	12 (7.2%)
Employed	
Yes	47 (28.8%)
No	116 (71.2%)
Need a caregiver	
Yes	110 (65.5%)
No	58 (34.5%)
Clinical relevant conditions	
Underlying chronic illness	
Yes	107 (63.7%)
No	61 (36.3%)
Time since Diagnosis	28.37±29.17
Relapsed disease	
Yes	39 (23.4%)
No	128 (76.6%)
Active cancer treatment	
Yes	127 (75.6%)
No	41 (24.4%)
Sleeping hours (<7 hours)	
Yes	71 (42.3%)
No	97 (57.3%)
Weight loss (mean per Kg)	1.14±6.92
ECOG-PS	
0,1	106 (59.6%)
2,3,4	62 (40.4%)
Infection	
Yes	21 (12.5%)
No	147 (87.5%)
Poor appetite	
Yes	101 (60.1%)
No	67 (39.9%)
Biomedical parameters	
Hb (Female <11 g/dl, Male < 13.5 g/dl)	11.08±2.22
WBCs (>11;10e9/L)	5.79±4.20
Sodium (<135 mmol/L)	137.45±2.91
Creatinine (>90;umol/L)	64.53±31.52
Calcium (>2.5;mmol/L)	2.30±0.20
Potassium (<3.4;mmol/L)	3.88±0.48
AST (>34;U/L)	29.51±26.88
Total Bilirubin (>20;umol/L)	11.33±12.87
ALT (>55;U/L)	37.10±36.29
LDH (>220;U/L)	318.30±175.65

CSF was present in 89 (53%) patients with a mean of 6.66±2.02, and 130 (77.4%) of the patients reported CRF with a mean severity score of 5.12±2.86. The four levels of CRF showed that the majority (34.6%) of patients suffered from severe CRF, followed by moderate CRF 44 (33.9%) (Table 2).

**Table 2:** Prevalence of CSF and CRF (n=168). CSF- Clinically significant fatigue; CRF- Cancer related fatigue

	n (%)	Mean ±SD
Clinically significant fatigue		
Yes	89 (53)	6.66±2.02
No	79 (47)	0.92±1.07
Fatigue by (severity)	130 (77.4)	5.12±2.86
Mild	41 (31.5)	1.78±0.82
Moderate	44 (33.9)	4.89±0.84
Severe	45 (34.6)	8.40±1.09

Table 3 shows the univariate and multivariate associations of CSF in the study population. In the univariate analysis, increasing age, relapsed disease, losing weight, and poor appetite were significantly associated with a high prevalence of CSF (p <0.05). By multivariate analysis, poor appetite was the only significant factor associated with CSF. Patients with poor appetite were 3.02 folds more likely to exhibit CSF than patients who have a good appetite, and thus increasing the prevalence of CSF (p <0.001).

**Table 3:** Univariate analysis for CSF (≥ 3/10) and independent predictors of CSF by multivariate logistic regression analyses (n=168). Data presented either as number and percentage or mean and standard deviation *Significant at p-value <0.005. ECOG-PS- Eastern Co-operative Oncology Group performance status; AST- aspartate transaminase, ALT- alanine aminotransferase, LDH- lactate dehydrogenase

	CSF group 89 (53)	Univariate analysis OR [95% CI]	Multivariate analysis OR [95% CI]
Age (mean±SD)	46.97±19.59	0.98 [0.97-0.99]*	0.99 [0.97-1.00]
Gender (Male)	54 (52.4%)	1.06 [0.57-1.97]	-
Marital Status			
Single	23 (46.9%)	1	-
Married	51 (52%)	3.39 [0.82-14.06]	
Divorced	5 (62.5%)	2.76 [0.71-10.83]	
Widowed	9 (75%)	1.80 [0.26-12.50]	
Employed	22 (46.8%)	1.45 [0.73-2.86]	-
Needs a caregiver (Yes)	62 (56.4%)	1.48 [0.78-2.81]	-
Underlying chronic illness (Yes)	56 (52.3%)	0.93 [0.49-1.75]	-
Time since diagnosis	29.47±28.30	0.99 [0.98-0.01]	-
Relapsed disease	26 (66.7%)	2.13 [1.06-4.51]*	1.83 [0.82-4.06]
Active cancer treatment	70 (55.1%)	0.33 [0.35-1.43]	-
Sleeping hours (<7 hours)	41 (57.7%)	0.92 [0.39-1.33]	-
Weight loss (mean per Kg)	2.38±7.80	1.07 [1.01-1.13]*	1 [0.95-1.07]
ECOG-PS (2,3,4)	36 (58.1%)	1.38 [0.74-2.61]	-
Infection (Yes)	12 (57.1%)	1.21 [0.48-3.05]	-
Poor appetite (Yes)	64 (63.4%)	2.91 [1.53-5.51]*	3.02 [1.33-6.85]*
Hb (Female <11 g/dl, Male < 13.5 g/dl)	10.75±2.30	1.16 [0.99-1.34]	-
WBCs (>11;10e9/L)	5.67±4.29	1.02 [0.94-1.09]	-
Sodium (<135 mmol/L)	137.48±3.02	0.99 [0.89-1.11]	-
Creatinine (>90;umol/L)	63.12±31.29	1.00 [0.99-1.01]	-
Calcium (>2.5;mmol/L)	2.28±0.17	2.96 [0.49-17.94]	-
Potassium (<3.4;mmol/L)	3.87±0.14	1.14 [0.59-2.19]	-
AST (>34;U/L)	31.55±27.11	0.99 [0.98-1.01]	-
Total Bilirubin (>20;umol/L)	11.57±13.19	0.99 [0.97-1.02]	-
ALT (>55;U/L)	38.49±32.37	0.99 [0.99-1.01]	-
LDH (>220;U/L)	309.05±141.72	1.00 [0.99-1.00]	-

**Figure 1:**
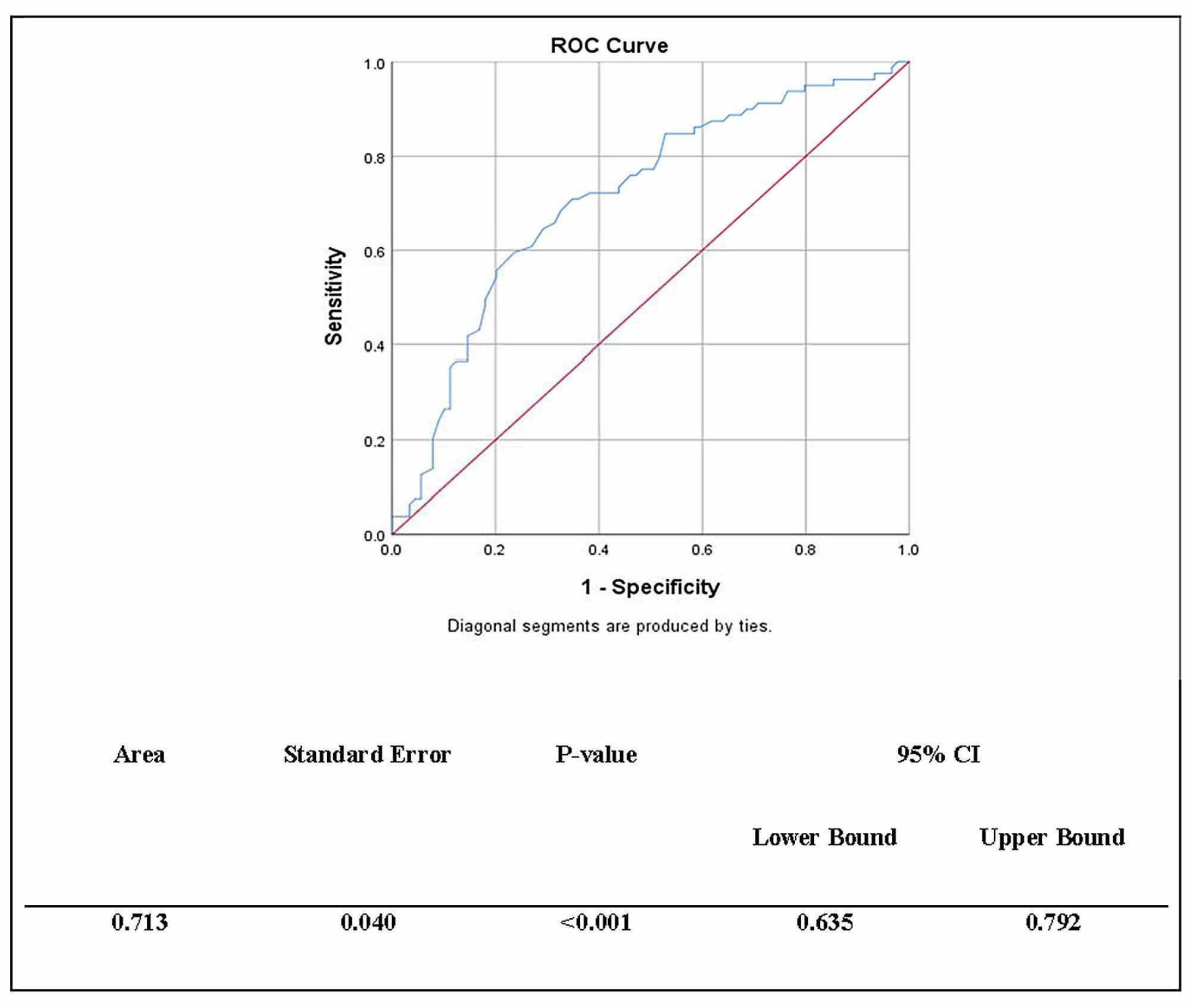
Receiver operating characteristic curve for FACT-Leu scale. FACT-Leu- Functional Assessment of Cancer Therapy-Leukemia; ROC curve- receiver operating characteristic curve; CI- Confidence interval

Figure 1 displays the ROC curves for the FACT-Leu scale. The ROC curve (0.713) was statistically significant, p<0.003. The cutoff point for FACT-Leu scale was found to be 126 out of 174 as the optimal cutoff point for good HRQoL. In the CSF group, 71 (79.8%) had poor HRQoL, while 35 (44.3%) of the non-CSF indicated poor HRQoL (Table 4).

**Table 4:** Distribution of good (FACT-Leu ≥126) and poor (FACT-Leu <126) HRQoL among CSF and non-CSF patients. *Significant at p-value <0.05 FACT-Leu- Functional Assessment of Cancer Therapy-Leukemia; HRQoL- Health-related quality of life; CRF- Cancer-related fatigue; CSF- clinically significant fatigue

	n(%)	Good HRQoL (n=62)	Poor HRQoL (n=106)	p-value
CSF group	89 (53)	18 (20.2%)	71 (79.8%)	<0.001*
Non-CSF group	79 (47)	44 (55.7%)	35 (44.3%)	

A statistically significant difference in the mean HRQoL was found between the CSF 104.89±28.82 and non-CSF 125.76±26.71 groups (p<0.001); however, both groups had a poor HRQoL (FACT-Leu <126). Moreover, in the 4 HRQoL domains, there were statistically significant differences in the HRQoL between the patients with CSF and non-CSF, except in the social well-being (p=0.239) (Table 5).

**Table 5:** Mean FACT-Leu scale (HRQoL) and subscales. *Significant at p-value <0.05 FACT-Leu- Functional Assessment of Cancer Therapy-Leukemia; HRQoL- Health-related quality of life

	Total Mean± SD	non-CSF group Mean± SD	CSF group Mean± SD	P-value
Total FACT-Leu scale	114.70±29.67	125.76±26.71	104.89±28.82	<0.001*
FACT-Leu domains				
Physical well-being (0-28)	16.40±7.76	19.85±7.15	13.38±7.02	<0.001*
Social well-being (0-28)	20.24±5.80	19.68±6.72	20.74±4.83	0.239
Emotional well-being (0-24)	17.26±5.18	18.72±4.45	15.97±5.45	<0.001*
Functional well-being (0-28)	17.70±6.90	19.03±6.92	16.55±6.70	0.021*
LEUS (0-68)	43.40±13.88	49.22±11.47	38.25±13.85	<0.001*

The multivariate analysis showed that patients who were dependent form caregivers were 3.13 folds more likely to exhibit good HRQoL than patients who are independent on a caregiver. Moreover, patients who have non-CSF were 5.22 folds more likely to exhibit good HRQoL than patients who have CSF (p <0.001) (Table 6).

**Table 6:** Independent predictors of good HRQoL (n=168). *Significant at p-value <0.05 ECOG-PS- Eastern Co-operative Oncology Group performance status; AST- aspartate transaminase, ALT- alanine aminotransferase, LDH- lactate dehydrogenase

	Good HRQoL (FACT-Lue ≥126)	Univariate analysis OR [95% CI]	Multivariate analysis OR [95% CI]
Age	40.05±16.64	0.98 [0.97-0.99]*	0.99 [0.97-1.01]
Gender (Male)	27 (26.2)	1.24 [0.65-2.37]	-
Marital Status			
Single	20 (40.8)	1	-
Married	38 (38.8)	3.45 [0.68-17.45]	
Divorced	2 (25)	3.17 [0.66-15.25]	
Widowed	2 (16.7)	1.17 [1.84-15.13]	
Non-employed	25 (53.2)	2.43 [1.21-4.85]*	0.74 [0.32-1.72]
Dependent form caregiver	32 (55.2)	3.28 [1.69-6.39]*	3.13 [1.33-2.44]*
Underlying chronic illness (No)	25 (41)	1.31 [0.69-2.51]	-
Time since Diagnosis	32.89±35.46	1.01 [0.99-1.02]	-
Relapsed disease (No)	53 (41.4)	2.36 [1.03-5.37]*	1.32 [0.51-3.44]
Active cancer treatment (Yes)	15 (36.6)	0.98 [0.47-2.04]	
Sleeping hours (>7 hours)	42 (43.3)	1.95 [1.01-3.75]*	2.14 [0.98-4.69]
Weight loss (mean per Kg)	-0.60±6.24	1.02 [0.97-1.07]	-
ECOG-PS (0,1)	44 (41.5)	1.74 [0.89-3.39]	-
Infection (No)	59 (40.1)	4.02 [1.13-14.27]*	3.56 [0.89-14.32]
Poor appetite (No)	28 (41.8)	1.41 [0.75-2.68]	-
Non-CSF	44 (55.7)	0.21 [0.10-0.40]*	5.22 [2.44-11.19]*
Hb (Female <11 g/dl, Male < 13.5 g/dl)	11.36±2.23	1.10 [0.95-1.27]	-
WBCs (>11;10e9/L)	5.03±3.58	0.93 [0.85-1.01]	-
Sodium (<135 mmol/L)	137.68±2.84	1.04 [0.93-1.17]	-
Creatinine (>90;umol/L)	65.60±26.11	1.00 [0.99-1.01]	-
Calcium (>2.5;mmol/L)	2.31±0.12	2.25 [0.41-12.33]	-
Potassium (<3.4;mmol/L)	3.92±0.38	1.25 [0.64-2.45]	-
AST (>34;U/L)	29.76±32.66	1.00 [0.99-1.01]	-
Bilirubin Total (>20;umol/L)	9.64±7.37	0.98 [0.95-1.01]	-
ALT (>55;U/L)	36.98±43.75	1.00 [0.99-1.01]	-
Lactate Dehydrogenase (>220;U/L)	296.28±198.42	0.99 [0.99-1.00]	-

## Discussion

The current study findings highlight mainly two aspects of leukemia patients’ care, fatigue, and HRQoL. Our results showed that leukemia patients had a high burden of CRF as reported by 77.4% of the study participants with a mean score of 5.12±2.86. Overall, this prevalence estimate indicates that fatigue is prevalent among leukemia patients and is consistent with previously reported estimates among leukemia patients, which have varied between 27% and 92% [9,19,20].

The majority of our study participants had either severe or moderate CRF; hence, 68.4% of the patients were identified as having CRF of a severity level that was considered CSF with a mean score of 6.66±2.02. A comparable result of CSF prevalence (62%, weighted proportion) was reported by Alibhai et al. (n=13) [20]. However, our reported prevalence and mean of CSF were higher than the findings reported by Wang et al., (2002) findings which have shown that 53.5% (weighted proportion) leukemia patients (n=106) had CSF with a mean score of 6.51±2.34 [21]. Lacourt et al. has reported 41.8% (weighted proportion) of CSF among acute myeloid leukemia (n=33) [22]. Furthermore, Romito et al. (2007) have reported a similar proportion (63.4%) of general cancer patients experienced CSF [8].

Our study findings indicated that ageing was not a predictor of CSF, which was consistent with previous studies in haematology malignancy patients [20,21]. In this study, other socio-demographic characteristics, including gender, marital status, and employment, need for a caregiver, and monthly income were also not predictive variables of CSF, which were consistent with Wang’s (2002) study [21]. 

In this study, evidence of chronic illness, weight loss, disease duration, active cancer treatment, ECOG-PS status, infection, and difficulty in sleeping (<7 hours) was not associated with CSF. A previous study indicated that the high prevalence and exacerbation of fatigue severity in cancer patients are demonstrated by disease progression [21]. However, ECOG-PS was not retained as a predictor of fatigue in our study.

Sleep and disturbance CSF have been documented as concurrent symptoms in leukemia patients [22]. Inconsistence with our results, previous literature demonstrated that insufficient sleep was significantly associated with CSF [21]. Furthermore, our results were consistent with earlier reports that poor appetite predicts and intensify CSF in patients with leukemia [23].

The association of anemia with CSF is controversial. Anemia could be attributed to the cancer disease itself, the myelosuppressive treatment, blood loss, or bone marrow infiltration [24]. In this study, anemia was not a predictor of CSF. Similarly, some studies have demonstrated that anemia is not associated with CSF [20,21]. The reason that anemia was not a predictor of fatigue in this study is most probably that anemia was transient and treated supportively, which might limit the effect of this variable on CSF among the study participants.

Fatigue was reported as a significant predictor of impaired HRQoL and poor survival in leukemia patients [20]. Fatigue can adversely disturb patients’ adherence to the treatment regime and can be dose-limiting [25]

The ROC analysis showed that the cut-off score for good HRQoL in our study sample (FACT-Lue) was ≥126 of 174. Overall, HRQoL mean score (104.89±28.82) assessed by FACT-Leu was below the optimal cut-off score indicating poor HRQoL among our leukemia patients. The results of HRQoL employing FACT-Leu reported in the literature were varied. These variances could be attributed to measuring a specific leukemia disease instead of all leukemia diseases, sample size, the phase of the disease, and type of treatment and study settings. In our discussion, we will compare our findings with studies using the FACT-Leu scale to eliminate measurement bias. Mamolo et al. found that adults with newly diagnosed or relapsed/refractory de novo Acute Myeloid Leukemia (AML) (n=68) had a poor HRQoL (FACT-Leu score: 100±24.0) [26]. Albrecht et al. had reported the HRQoL experienced by newly diagnosed AML patients during chemotherapy induction was at baseline and up to six weeks during the chemotherapy (FACT-Leu score: 112.39±22.11) [27]. Tinsley et al. had reported as well a poor HRQoL among AML patients during the intensive treatment (FACT-Leu score: 118.4±24.9) and non-intensive treatment (FACT-Leu score: 120.3±24.8) [28]. Moreover, Kayastha et al. indicated that relapsed AML patients had poor HRQoL (FACT-Leu score=113.4±37.0) [29]. Remarkably, the FACT-Leu mean score of the CSF (104.89±28.82) and non-CSF (125.76±26.71) patients indicated a poor HRQoL (<126 of 174) among our study participants.

Striving with physical activity and psychosocial aspects, living with ambiguity, fear about future and disease relapse and effect on daily life are evident in hematologic malignancies patients, including leukemia. In the present study, FACT-Leu subscales mean scores differentiated showed that the patients with CSF had worst HRQoL than patients with non-CSF, except with SWB. Similar results were observed in the study performed by Kayastha et al., in the PWB score [29]. However, our study patients showed better values in the SWB, EWB, FWB, and LEUS scores in comparison to Kayastha et al., and Mamolo et al., [26,29]. The higher level of SWB in the current study may perhaps reflect the levels of social support given to leukemia patients at the study settings. Overall, these results figure out the need to enhance the whole status of patients with Leukemia, in particular, those who indicated physical impairment.

Several variables have been identified in the multivariate regression model. A caregiver plays a crucial role in patients’ management of their illness. They are often the primary source of physical, social, and emotional support for the patient. Our results support this by asserting that patients who are independent of caregivers’ support are less likely to have better HRQoL than patients who have a caregiver. Distressing symptoms experienced by leukemia patients like disturbed sleeping disrupt their HRQoL. Therefore, sufficient sleeping hours predict better HRQoL. Fatigue is associated with declines in all subscales of FACT-Leu scale and adversely interferes with the physical and psychosocial domains of HRQoL [30]. Because fatigue among cancer patients, is inevitable, extensive efforts and strategies shall be made to reduce fatigue level to the mild levels. Identification of fatigue and HRQoL could lead to the development of supportive care programs based on a patient-centred approach and experience.

Several limitations should be considered when interpreting our data. The study sample size was modest. The cross-sectional design does not provide definite causation between fatigue and HRQoL among leukemia patients. Our study was conducted in a single institution; although the largest in Saudi Arabia, our data generalizability might be threatened. Our study did not identify fatigue severity and patients’ quality of life-based on the type of leukemia or type of treatment. 

## Conclusions

CSF is widespread among patients with leukemia. It is essential to identify the prevalence and the predictors of CSF and HRQoL to develop a patient-centred supportive care program. Our study findings support the importance of adapting NCCN guidelines to screen, evaluate, and manage CSF. Oncologists should be aware of CSF and address it throughout the continuum of leukemia treatment.
